# Melatonin Suppresses LPS-Induced Oxidative Stress in Dendritic Cells for Inflammatory Regulation via the Nrf2/HO-1 Axis

**DOI:** 10.3390/antiox11102012

**Published:** 2022-10-11

**Authors:** Tao Qin, Danni Feng, Bangyue Zhou, Lirong Bai, Yinyan Yin

**Affiliations:** 1 College of Veterinary Medicine, Yangzhou University, Yangzhou 225009, China; 2 Jiangsu Co-Innovation Center for the Prevention and Control of Important Animal Infectious Disease and Zoonoses, Yangzhou University, Yangzhou 225009, China; 3 College of Medicine, Yangzhou University, Yangzhou 225009, China; 4 Jiangsu Key Laboratory of Experimental and Translational Non-Coding RNA Research, Yangzhou University, Yangzhou 225009, China

**Keywords:** melatonin, acute lung injury, oxidative stress, dendritic cells, inflammation

## Abstract

Melatonin, an indoleamine synthesized in the pineal gland of mammals, is a natural bioactive compound with powerful antioxidant and anti-inflammatory properties. Here, we evaluated whether melatonin has the capacity to moderate the oxidative stress of dendritic cells (DCs) for inflammatory control in an acute lung injury (ALI) model. Our findings showed that melatonin remarkably inhibited total nitric oxide synthase (T-NOS) activity, nitric oxide (NO) production, intracellular reactive oxygen species (ROS) levels, and lipid peroxidation (MDA detection) levels in both an LPS-induced murine ALI model and LPS-induced DCs. Meanwhile, the reduced glutathione (GSH) level and the GSH/GSSG ratio were recovered. In addition, antioxidant enzymes, such as glutathione peroxidase (GPx), catalase (CAT), and superoxide dismutase (SOD), were increased in these processes. Moreover, melatonin also inhibited the LPS-induced secretions of IL-1β, IL-6, and TGF-β in vivo and in vitro. Finally, we found that the nuclear factor erythroid 2-related factor 2 (Nrf2)/heme oxygenase 1 (HO-1) axis was required in the inhibition of LPS-induced oxidative stress in DCs by melatonin. Altogether, these data indicate that melatonin strongly suppresses the LPS-induced oxidative stress in DCs, which is a promising DC-targeted strategy via inflammatory control for ALI treatment.

## 1. Introduction

Oxidative stress stems from the imbalance between pro-oxidants and antioxidants in favor of the oxidants, leading to a disruption of physiological redox signaling and control, which damages cellular functions [1,2]. Notably, a great number of reactive oxygen species (ROS) and reactive nitrogen species (RNS) often accompany each other [3]. Oxidative stress has been reported to directly or indirectly participate in a wide range of diseases, such as diabetes, atherosclerosis, Alzheimer’s disease, cancer, sepsis, and systemic inflammatory response syndrome (SIRS). Except for directly oxidizing membrane lipids, structural proteins, and DNA for contributing to diseases, oxidative stress, as a secondary contributor, promotes disease progression by disturbing multiple signaling pathways to aggravate inflammatory injuries [4]. Therefore, it is necessary to develop antioxidant treatment strategies to control the progression of various diseases induced by oxidative stress.

Acute lung injury (ALI) is defined as an inflammation-mediated severe pulmonary disease that results in diffuse pulmonary interstitial and parenchymal edema, which may progress to potential acute respiratory distress syndrome (ARDS) [5]. Recently, a study showed that the incidence rate of ALI was 1~4% and the case fatality rate was 22~65% [6]. Lipopolysaccharide (LPS), a critical component of Gram-negative bacteria, is closely associated with ALI [7,8]. Recently, SARS-CoV-2 infection was reported to accelerate the pathophysiological processes to develop multiple organ dysfunction with high mortality in ALI patients [9]. The formation of oxidative stress and the resulting inflammation are thought to be important mechanisms in ALI [10]. Oxidative stress enhances the recruitment and activation of inflammatory cells, leading to an amplified inflammatory response cascade and thereby cellular injury [11]. More reports have supported the idea that oxidants and oxidative injury play an important role in the pathogenesis of ALI/ARDS. For instance, a high level of H_2_O_2_ was found in the exhaled breath condensate of patients with ARDS [12]. Additionally, oxidatively modified proteins appeared in overload, whereas antioxidant molecules showed an insufficient quantity in the bronchoalveolar lavage fluid of ARDS patients [13,14]. Therefore, how to rebuild the oxidant/antioxidant balance to control oxidative stress in ALI needs to be addressed.

Dendritic cells (DCs) are not only classic antigen-presenting cells but also play an important role in the maintenance and regulation of immune homeostasis toward immune activation or immune tolerance based on the type of stimulation [15]. In the lungs, DCs show a key regulatory function in the immune response network. Previous studies have indicated that mature lung conventional DCs (cDCs), an immune activation state, were involved in acute lung inflammation and pathological injury progress in ALI [16,17], implying that DC-targeted immune regulation strategies are prospective for ALI therapy. Especially, a study showed that the manipulation of DCs toward tolerance can attenuate the ALI [18], which provides the possibility to alleviate ALI by regulating DCs. Notably, DCs also can regulate the balance of oxidative stress to influence inflammatory responses. Our previous studies found that some antioxidants, such as BP5 and astaxanthin, provided a strong antioxidant ability for inflammatory control in LPS-induced DCs for sepsis treatment [19,20]. Therefore, DC-targeted oxidative stress regulation is a promising strategy for the treatment of ALI.

Melatonin, also known as N-acetyl-5-methoxytryptamine, is a tryptophan derivative that is structurally related to other important substances such as serotonin and is naturally produced by the pineal gland in mammals [21,22]. Melatonin mainly regulates body temperature and reproductive hormone levels. Recently, it was reported to have strong antioxidant and anti-inflammatory characteristics [23]. In radiation or LPS-induced lung injury models, melatonin displayed a protective effect via down-regulating the NLRP3 inflammasome [24,25], implying that melatonin showed a good anti-inflammatory ability for ALI treatment. Nevertheless, how melatonin globally regulates the level of oxidative stress in LPS-induced ALI in vivo and in LPS-induced DCs in vitro remains unclear. Here, the antioxidant ability of melatonin was systematically evaluated, which provides evidence that a DC-targeting regulation strategy could be effectively applied in ALI treatment.

## 2. Materials and Methods

### 2.1. Ethics Statement

Animal care and experimental procedures in the study were in compliance with the laboratory animal welfare and ethics guidelines of the Jiangsu Administrative Committee for Laboratory Animals and have been approved by the Jiangsu Administrative Committee for Laboratory Animals (permission number: SYXK(SU)2017-0044).

### 2.2. Reagents and Antibodies

Melatonin (molar mass: 232.28 g·mol^−1^), cobalt protoporphyrin (CoPP, an inducer of HO-1 [15]), and Lipopolysaccharides (from *Escherichia coli* O55: B5; code: L2880) were purchased from Sigma-Aldrich (St. Louis, MO, USA). RPMI 1640 medium and fetal bovine serum (FBS) were sourced from Thermo Fisher Scientific (Waltham, MA, USA). Streptomycin and penicillin were obtained from Invitrogen (Grand Island, NY, USA). Recombinant mouse granulocyte-macrophage colony-stimulating factor (GM-CSF) and interleukin-4 (IL-4) were obtained from Peprotech (Rocky Hill, CT, USA). We obtained the following reagents from Beyotime Biotechnology (Shanghai, China), including nitric oxide (NO) assay kit, catalase (CAT) analysis kit, reactive oxygen species (ROS) assay kit, bicinchoninic acid (BCA) protein assay kit, cell and tissue lysis buffer for nitric oxide assay, and RIPA lysis buffer. The following detection kits, including total nitric oxide synthase (T-NOS) assay kit, malondialdehyde (MDA) assay kit, superoxide dismutase (SOD) assay kit, glutathione peroxidase (GPx), and total glutathione/oxidized glutathione (T-GSH/GSSG) assay kit were from Jiancheng Bioengineering Institute (Nanjing, Jiangsu, China). Interleukin-1β (IL-1β), transforming growth factor-β (TGF-β) and interleukin-6 (IL-6) ELISA kits were obtained from Multi Sciences (Hangzhou, Zhejiang, China). Tin protoporphyrin IX (SnPP, an inhibitor of HO-1 [15]) was obtained from MedChemExpress (Monmouth Junction, NJ, USA). Alexa Fluor 647 HO-1 or respective isotype was purchased from Abcam (Cambridge, Cambs, UK). PE-Nrf2 or respective isotype was sourced from Cell Signaling Technology (Boston, MA, USA).

### 2.3. Bone Marrow-Derived DCs (BMDCs) Culture and Treatment

Four- to six-week-old specific pathogen-free (SPF) C57BL/6 mice were acquired from the Laboratory Animal Center of Yangzhou University (Yangzhou, Jiangsu, China). BMDCs were isolated and cultured from myeloid precursor cells, following our previous improved method [15]. Briefly, myeloid precursor cells were flushed from the medullary cavity of the tibias and femurs of mice and resuspended in RPMI 1640 and 10% FBS complete medium with 10 ng/mL GM-CSF and IL-4, 1% streptomycin and penicillin. To isolate the purified immature BMDCs, non-adherent cells were gently discarded on day 3. After 6 days of culturing, we collected loosely adherent and non-adherent cells for sub-culturing overnight. On day 7, 90% or more of the CD11c^+^ non-adherent cells were treated with melatonin and/or LPS.

### 2.4. Establishment of LPS-Induced ALI Murine Model and Melatonin Intervention

SPF C57BL/6 mice (aged 6–8 weeks old, weight 18–22 g) were used in this study. The establishment of the ALI murine model was according to previous studies with minor modifications [26]. The murine ALI model was induced by the intratracheal injection of LPS with a dosage of 20 mg/kg. Briefly, after mice were anesthetized, LPS was injected directly into the trachea through the tracheostomy to induce the murine ALI model, and the incision was glued with tissue adhesive. The mice were put back into the cage until fully awake. Mice were randomly divided into 4 groups (*n* = 5), including the phosphate-buffered saline (PBS) (intratracheally, i.t.) group, melatonin (30 mg/kg [25]; intraperitoneally, i.p.) group, ALI group, and ALI + melatonin (30 mg/kg; i. p. for 12 and 1 h (h) before LPS administration).

### 2.5. T-NOS Activity and NO Measurement

To detect T-NOS activity in vivo, the lung tissue of mice was homogenized with PBS at a ratio of 10% at 6 h after LPS or PBS challenge and then centrifuged (2500 rpm, 4 °C, 10 min). In vitro, DCs were lysed with RIPA buffer and centrifuged to remove debris to obtain cellular proteins after LPS and/or melatonin stimulation for 24 h. The T-NOS activity of the lung supernatant and DC proteins were detected by the T-NOS assay kit and measured by using a microplate reader (BioTek Synergy 2, Winooski, VT, USA) at 530 nm. For NO detection, at 6 h after ALI induction in vivo, the fresh lung tissue was collected and lysed with cell and tissue lysis buffer according to the method of the NO detection kit. In vitro, DCs were plated in 24-well plates with 1 × 10^6^ cells/mL, after treatment with different concentrations of melatonin in the presence or absence of LPS (100 ng/mL) for 24 h, and the cultured cell supernatants were collected. According to the manufacturer’s instructions, the levels of NO in the lung tissue and culture medium were measured on the microplate reader (BioTek Synergy 2, Winooski, VT, USA) at 540 nm.

### 2.6. ROS Level Detection

DCs were primed with LPS and/or melatonin for 24 h, then incubated with 10 µM 2’,7’ dichlorofluorescein diacetate (DCFH-DA) for 20 min at 37 °C in dark conditions. Cells were washed three times with cold PBS. Intracellular ROS generation was determined using a flow cytometer (BD FACSVerse, Franklin Lakes, NJ, USA) and analyzed by FlowJo software version 10.6.2 (Tree Star, Ashland, OR, USA).

### 2.7. MDA Content Detection

Lung tissue samples were homogenized to a 10% ratio with ice-cold PBS, then centrifuged to obtain the supernatant (2500 rpm, 4 °C, 10 min). In addition, after culturing for 24 h, the DCs were centrifuged (2000 rpm, 4 °C, 10 min) and the supernatant was collected. The MDA levels in the supernatant were measured according to the instructions of the commercial kit.

### 2.8. Glutathione (GSH) and the Oxidized Glutathione (GSSG) Analysis

According to the operating procedure of the assay kits, the lung tissue or DCs were dissolved in extraction buffer to form a 20% homogenate and then centrifuged (3500 rpm, 4 °C, 10 min). The T-GSH content and GSSG content in the supernatant were determined based on the method of the assay kits and measured by using BioTek Synergy 2 (Winooski, VT, USA) at 405 nm for the analysis of T-GSH and GSSG levels. The GSH content was obtained by subtracting the 2 × GSSG value from the T-GSH value. The GSH/GSSG ratio was calculated with reference to our previous method [19].

### 2.9. GPx, CAT, and SOD Detection

The fresh lung tissue samples were homogenized in ice-cold PBS to make a 10% tissue homogenate and were centrifuged at 2500 rpm for 10 min at 4 °C. The supernatant was extracted to detect GPx activity, CAT activity, and SOD activity according to the method of the assay kits. Similarly, after DCs were treated with different groups for 24 h, the protein was extracted for detecting GPx activity, CAT activity, and SOD activity by the kits.

### 2.10. Cytokines Measurement

Bronchoalveolar lavage fluid (BALF) samples were harvested at 6 h after PBS or LPS i.t., and centrifuged for 10 min with 1500 rpm at 4 °C to extract the supernatants. In vitro, the DC culture supernatants were collected after 24 h for further analysis. The inflammatory cytokines such as IL-1β, IL-6, and TGF-β in the collected fluid were tested by using the corresponding ELISA kits following the manufacturer’s instructions.

### 2.11. Statistical Analysis

All results were presented as the means ± SD and analyzed with SPSS 26.0 software (SPSS Inc, Chicago, IL, USA). To compare multiple groups, a one-way analysis of variance (ANOVA) was used to compare the variance between different groups. A *p*-value less than 0.05 was considered to be statistically significant.

## 3. Results

### 3.1. Melatonin Reduced the NOS Activity and NO Production in the LPS-Induced ALI Mice and LPS-Induced DCs

**Figure 1:**
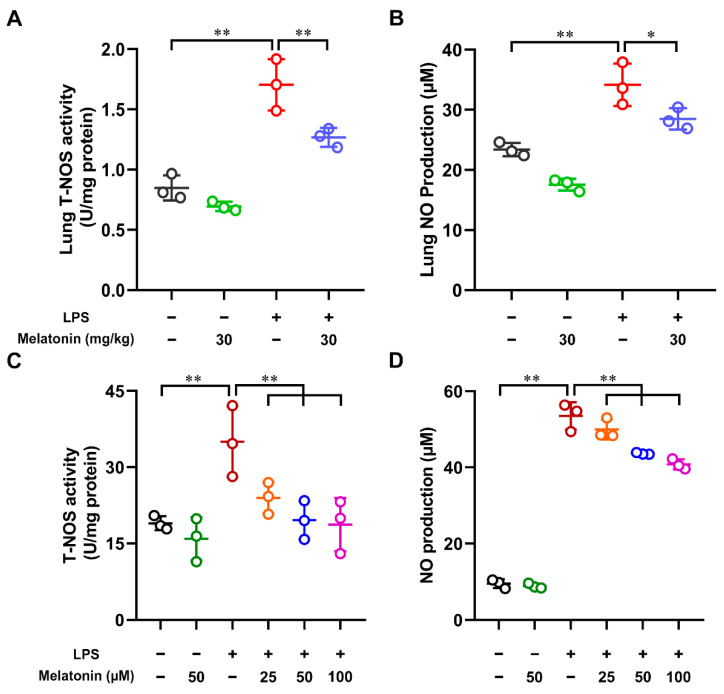
The effect of melatonin on the T-NOS activity and NO production in the lung tissue of LPS-induced ALI mice and in the LPS-induced DCs. (**A**,**B**) Melatonin was administrated intraperitoneally for 12 and 1 h before the intratracheal injection of LPS. Lung was harvested at 6 h after LPS challenge. The total nitric oxide synthase (T-NOS) activity (**A**) and nitric oxide (NO) level (**B**) in the supernatants of lung tissue homogenate were tested by using commercial kits. (**C**,**D**) DCs were treated with different concentrations of melatonin in the presence or absence of LPS (100 ng/mL) for 24 h; the DC supernatants were harvested and the DCs were lysed. The T-NOS activity (**C**) in DC lysates and NO level (**D**) in DC supernatants were measured according to Materials and Methods. The data shown represent the means ± SD from one of three independent experiments. Statistical significance is determined using variance (ANOVA). * *p* < 0.05, ** *p* < 0.01.

Nitrogen oxide (NO) is not only a marker of oxidative stress but is also a reactive molecule produced by nitric oxide synthase (NOS) enzymes in a variety of cells [27,28]. In order to detect the total nitric oxide synthase (T-NOS) activity and nitric oxide (NO) level in the model of acute lung injury (ALI), the lung tissue was collected and lysed at 6 h after LPS challenge. The T-NOS activity and NO production were measured by using commercial kits. The results showed that the T-NOS activity and NO level were markedly increased in the LPS-only group compared with those in the control group (*p* < 0.01). Furthermore, the mice were administrated with melatonin i.p. before LPS i.t. We found that both the T-NOS activity and NO level were strongly decreased in the LPS plus melatonin group compared with those in the LPS alone group (*p* < 0.05, Figure 1A,B). Meanwhile, we also tested whether melatonin affected the T-NOS activity and NO production in the LPS-induced DCs. Significantly, the T-NOS activity and NO level were down-regulated by melatonin in a dose-dependent manner (*p* < 0.01, Figure 1C,D). Altogether, these results indicated that melatonin strongly suppressed the NOS activity and NO production in the LPS-induced ALI mice and LPS-induced DCs.

### 3.2. Melatonin Suppressed the ROS Level in the LPS-Induced DCs

**Figure 2:**
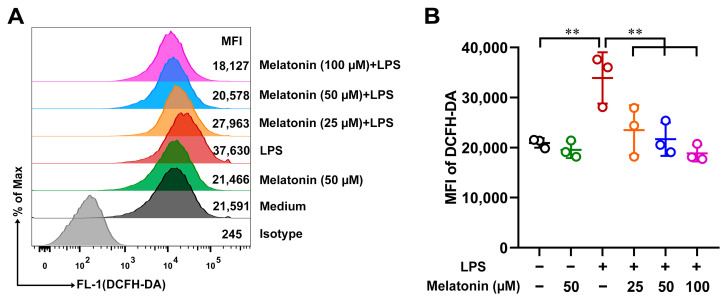
The effect of melatonin on ROS production in the LPS-induced DCs. (**A**) DCs were treated with different concentrations of melatonin in the presence or absence of LPS (100 ng/mL) for 24 h; DCs were incubated with DCFH-DA and detected by FCM. (**B**) The data shown represent the means ± SD from one of three independent experiments. Statistical significance is determined using variance (ANOVA). ** *p* < 0.01.

Under physiological conditions, ROS participate in the maintenance of cellular redox homeostasis to protect cells from oxidative stress. However, the overproduction of ROS results in oxidative stress and leads to lung damage [29]. To study whether melatonin could inhibit ROS production in the LPS-induced DCs, DCs were incubated with DCFH-DA and detected by flow cytometry (FCM). After treatment with LPS for 24 h, the ROS level in DCs was significantly up-regulated compared with those of the control (*p* < 0.01, Figure 2A,B). However, the ROS level in the LPS-induced DCs was strongly down-regulated by melatonin (*p* < 0.01, Figure 2A,B). These results suggested that melatonin treatment significantly suppressed the ROS level in the LPS-induced DCs.

### 3.3. Melatonin Inhibited Lipid Peroxidation in the LPS-Induced ALI Mice and LPS-Induced DCs

**Figure 3:**
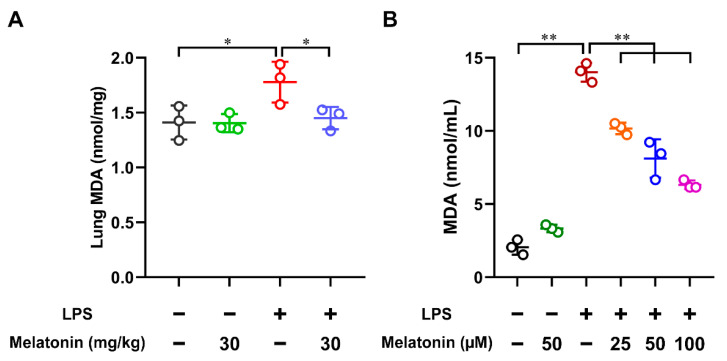
The effect of melatonin on lipid peroxidation in the lung tissue of LPS-induced ALI mice and in the LPS-induced DCs. (**A**) In the ALI model, lung tissue samples were harvested and homogenized at 6 h after LPS challenge. (**B**) DCs were stimulated with different concentrations of melatonin in the presence or absence of LPS (100 ng/mL) for 24 h and the DC supernatants were harvested. The MDA contents in the lung tissue lysate and DC supernatants were tested by using commercial kits. The data shown represent the means ± SD from one of three independent experiments. Statistical significance is determined using variance (ANOVA). * *p* < 0.05, ** *p* < 0.01.

MDA is the end-product of lipid peroxidation, and MDA formation acts as a marker for oxidative stress [30]. In order to detect the MDA content in the LPS-induced ALI mice, the lung tissue was harvested and homogenized at 6 h after LPS challenge. The results showed that the MDA contents were strongly increased in the LPS-only group compared with those in the control group (*p* < 0.05), while they were significantly suppressed after the administration of melatonin in the LPS-induced ALI mice (*p* < 0.05, Figure 3A). In addition, the MDA contents in the LPS-induced DCs were also examined. After LPS stimulation for 24 h, the MDA contents in the DC supernatants were significantly increased (*p* < 0.01), while they were strongly inhibited by the treatment of melatonin in a concentration-dependent manner (*p* < 0.01, Figure 3B). Accordingly, these observations indicated that melatonin treatment strongly inhibited lipid peroxidation in the LPS-induced ALI mice and LPS-induced DCs.

### 3.4. Melatonin Regulated the GSH, GSSG, and GSH/GSSG Ratio in the LPS-Induced ALI Mice and LPS-Induced DCs

**Figure 4:**
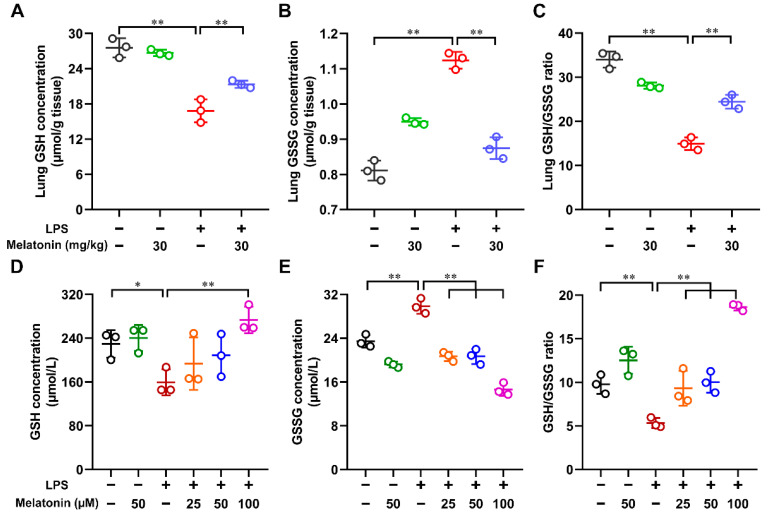
The regulation of melatonin on the GSH, GSSG, and GSH/GSSG ratio in the lung tissue of LPS-induced ALI mice and in the LPS-induced DCs. (**A**–**C**) In the ALI model, lung tissue samples were homogenized at 6 h after LPS challenge. (**D**–**F**) DCs were stimulated with different concentrations of melatonin in the presence or absence of LPS (100 ng/mL) for 24 h, then the DCs were harvested and lysed. The GSH content (**A**,**D**), GSSG content (**B**,**E**), and the ratio of GSH/GSSG (**C**,**F**) in the lung tissue and DC lysate were tested according to Materials and Methods. The data shown represent the means ± SD from one of three independent experiments. Statistical significance is determined using variance (ANOVA). * *p* < 0.05, ** *p* < 0.01.

Next, to study whether melatonin could regulate the reduced glutathione (GSH) level, oxidized glutathione (GSSG) level, and the ratio (GSH/GSSG), the lung tissue and DCs were lysed. These results showed that LPS markedly decreased the GSH level, increased the GSSG level, and reduced the ratio of GSH/GSSG in the lung tissue of ALI mice (*p* < 0.01). However, these effects were strongly reversed after the administration of melatonin (*p* < 0.01, Figure 4A–C). In vitro, after LPS stimulation for 24 h, the GSH level (*p* < 0.05) and the ratio of GSH/GSSG (*p* < 0.01) were decreased, the GSSG level (*p* < 0.01) was increased in DC lysate. Similarly, in vivo, melatonin strongly reversed those processes (*p* < 0.01, Figure 4D–F). Collectively, these results indicate that melatonin regulates the GSH, GSSG, and GSH/GSSG ratio in LPS-induced ALI mice and LPS-induced DCs.

### 3.5. Melatonin Improved the Activities of Antioxidant Enzymes in the LPS-Induced ALI Mice and LPS-Induced DCs

**Figure 5:**
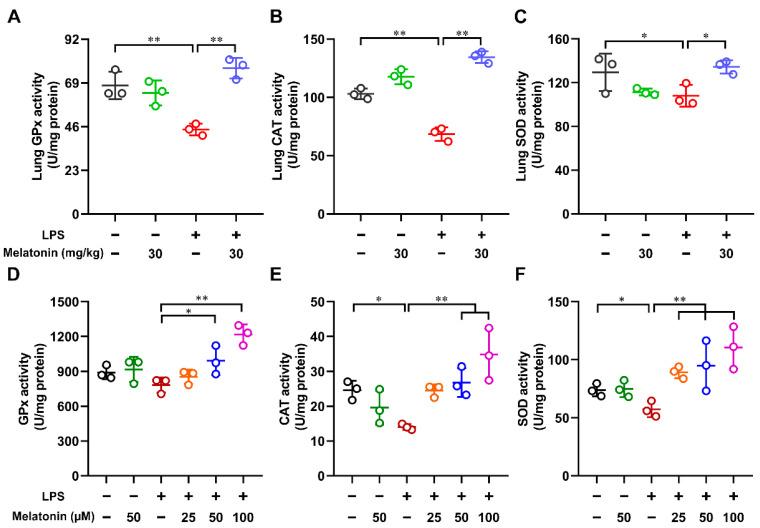
The effect of melatonin on the activities of antioxidant enzymes in the lung tissue of LPS-induced ALI mice and in the LPS-induced DCs. (**A**–**C**) In the ALI model, lung tissue samples were lysed at 6 h after LPS challenge. (**D**–**F**) DCs were treated with different concentrations of melatonin in the presence or absence of LPS (100 ng/mL) for 24 h and then were lysed. The activities of GPx (**A**,**D**), CAT (**B**,**E**), and SOD (**C**,**F**) in the lung tissue and cell lysate were tested according to Materials and Methods. The data shown represent the means ± SD from one of three independent experiments. Statistical significance is determined using variance (ANOVA). * *p* < 0.05, ** *p* < 0.01.

Antioxidative enzymes such as glutathione peroxidase (GPx), catalase (CAT), and superoxide dismutase (SOD) play a key role in the scavenging of those activated species such as superoxide and hydrogen peroxide to reduce oxidative stress [31]. Here, the lung tissue of LPS-induced ALI mice and LPS-induced DCs were lysed, and the activities of antioxidant enzymes (GPx, CAT, and SOD) were measured. As shown in Figure 5A–C, After LPS treatment alone, the activities of GPx, CAT, and SOD in the lung tissue of ALI mice were strongly reduced (*p* < 0.05). However, these processes were reversed by the administration of melatonin in the LPS-induced ALI mice (*p* < 0.05). As expected, melatonin increased the activities of GPx, CAT, and SOD in LPS-stimulated DCs in a dose-dependent manner (Figure 5D–F). These results suggested that melatonin treatment improved the activities of antioxidant enzymes in the LPS-induced ALI mice and LPS-induced DCs.

### 3.6. Melatonin Impaired the Cytokines Secretion in the LPS-Induced ALI Mice and LPS-Induced DCs

**Figure 6:**
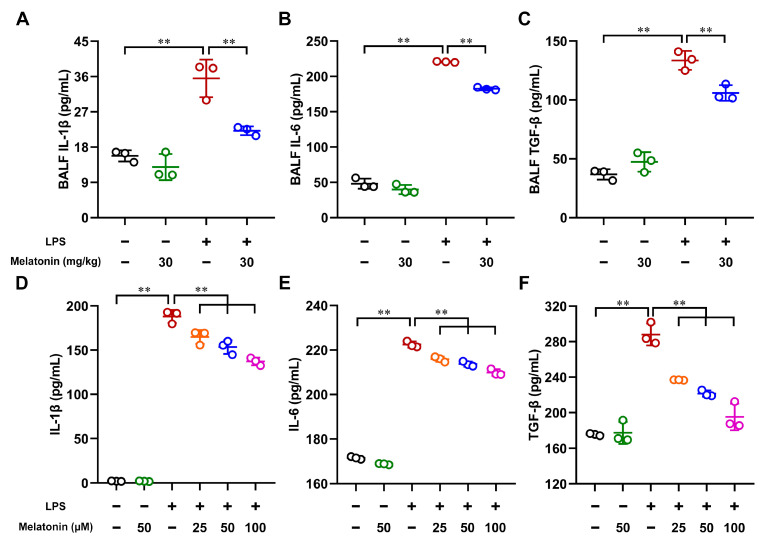
The effect of melatonin on the cytokine levels in the bronchoalveolar lavage fluid (BALF) of LPS-induced ALI mice and in LPS-induced DCs. (**A**–**C**) In the ALI model, BALF samples were collected at 6 h after LPS challenge. (**D**–**F**) DCs were treated with different concentrations of melatonin in the presence or absence of LPS (100 ng/mL) for 24 h, and then the DC supernatants were harvested. The secretion of IL-1β, IL-6, and TGF-β in the BALF and DC supernatants was measured by using commercial kits. The data shown represent the means ± SD from one of three independent experiments. Statistical significance is determined using variance (ANOVA). ** *p* < 0.01.

Next, to investigate whether melatonin could regulate cytokine secretion, the bronchoalveolar lavage fluid (BALF) of LPS-induced ALI mice and the supernatants of LPS-induced DCs were harvested. As shown in Figure 6A–C, the secretion of IL-1β, IL-6, and TGF-β was significantly increased in the LPS-only group compared with those in the control group (*p* < 0.01). However, these processes were strongly inhibited after the administration of melatonin in the LPS-induced ALI mice (*p* < 0.01). Furthermore, we also assessed the secretion of IL-1β, IL-6, and TGF-β in vitro. As expected, melatonin suppressed the cytokine secretion in the LPS-induced DCs in a dose-dependent manner (*p* < 0.01, Figure 6D–F). Therefore, these results indicate that melatonin impairs cytokine secretion in LPS-induced ALI mice and LPS-induced DCs.

### 3.7. Melatonin Attenuated Oxidative Stress in the LPS-Induced DCs via Nrf2/HO-1 Signaling Pathways

**Figure 7:**
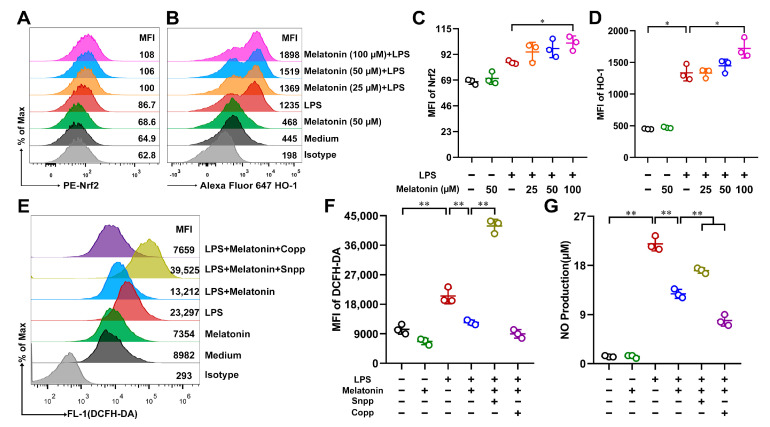
Regulation of melatonin on the Nrf2/HO-1 signaling pathways in the LPS-induced DCs. (**A**–**D**) DCs were treated with different concentrations of melatonin in the presence or absence of LPS (100 ng/mL) for 24 h, the treated DCs were labeled with PE-Nrf2 or Alexa Fluor 647 HO-1 antibody, and respective isotype for analyzing the protein expressions, after washing, the cells were detected by FCM. (**E**–**G**) DCs were stimulated with melatonin (50 μM) and/or LPS in the presence or absence of SnPP (25 μM) or CoPP (50 μM) for 24 h. (**E**,**F**) DCs were incubated with DCFH-DA and detected by FCM. (**G**) NO production in DC supernatants was measured according to Materials and Methods. The data shown represent the means ± SD from one of three independent experiments. Statistical significance is determined using variance (ANOVA). * *p* < 0.05, ** *p* < 0.01.

Heme oxygenase 1 (HO-1) plays a critical protective role in various insults-induced acute lung injuries (ALIs) through its strong anti-oxidant properties [32]. Nuclear factor-erythroid 2 related factor 2 (Nrf2) is a ubiquitous master transcription factor that up-regulates the antioxidant response element (ARE)-mediated expression of antioxidant enzymes and cytoprotective proteins. Nrf2 activation has been shown to be protective against lung injury [33]. To demonstrate whether melatonin might suppress oxidative stress via the Nrf2/HO-1 signaling pathways, the expressions of Nrf2 and HO-1 on DCs were analyzed by FCM. As shown in Figure 7A–D, melatonin strongly increased the expressions of Nrf2 and HO-1 in the LPS-induced DCs. We further investigated whether HO-1 played a vital role in the antioxidant effects induced by melatonin in the LPS-induced DCs. ROS levels (Figure 7E,F) and NO production (Figure 7G) were detected. The results showed that the antioxidant effects induced by melatonin were diminished when DCs were treated with tin protoporphyrin IX (SnPP, an inhibitor of HO-1) in the LPS-induced DCs, whereas cobalt protoporphyrin (CoPP, an inducer of HO-1) further aggravated the antioxidant effects of melatonin (Figure 7E–G). Therefore, melatonin treatment attenuated oxidative stress in the LPS-induced DCs via the Nrf2/HO-1 signaling pathways.

## 4. Discussion

**Figure 8:**
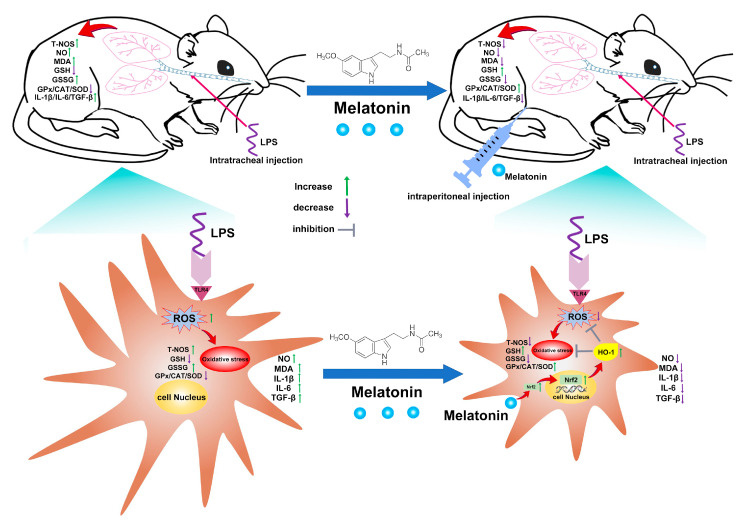
Schematic of proposed mechanism by which melatonin attenuates oxidative stress in ALI. Melatonin first activates the Nrf2/HO-1 signaling pathways and then inhibits oxidative stress in LPS-induced DCs, which is helpful for inflammatory control in ALI treatment.

Here, our work revealed the antioxidative properties of melatonin in LPS-induced DCs and mice, showing a promising DC-targeted oxidative stress regulation strategy for ALI treatment (Figure 8). The results suggested that melatonin remarkably inhibited T-NOS activity and NO production, intracellular ROS levels, and lipid peroxidation levels (MDA detection) in the LPS-induced murine ALI model and LPS-induced DCs. In addition, the GSH level, GSH/GSSG ratio, and antioxidant enzymes including GPx, CAT, and SOD were restored in these processes. Meanwhile, melatonin strongly suppressed the secretion of IL-1β, IL-6, and TGF-β inflammatory cytokines in vivo and in vitro. Furthermore, we found that the Nrf2/HO-1 axis was required in the antioxidation process of melatonin.

Nitric oxide (NO) in mammalian cells is produced by a multistep reaction route of L-arginine catalyzed by a family of NO synthases (NOS) [34]. There is evidence that NO production is increased particularly in humans with ALI or ARDS, as NO is clearly associated with the pathophysiology of ALI by modulating levels of inflammatory mediators [35,36]. The interaction between NO and ROS can promote inflammation progress, which is a major mechanism of NO production in ALI [36]. Meanwhile, ROS can induce lipid peroxidation with the polyunsaturated fatty acids of lipid membranes for further pathological processes [37]. Our results showed that melatonin remarkably inhibited T-NOS activity, NO production, and lipid peroxidation in the LPS-induced murine ALI model. In the DCs, we also found that not only the increases of T-NOS and NO were inhibited but high intracellular ROS levels and lipid peroxidation levels were also suppressed. Although lymphocytes and DCs reside in the lungs in relatively small numbers, they participate in the pathophysiology of ALI [38], because over-activated DCs may amplify inflammatory signals via crosstalk with downstream lymphocytes [39]. Therefore, DCs are ideal targets for ALI treatment.

GSH, a tripeptide, is an important endogenous antioxidant produced by host cells that can provide protection against ROS such as free radicals and peroxides by minimizing disulfide bond formation to the cysteine residues on cytoplasmic proteins. GSH has been shown to be converted into GSSG to execute antioxidant functions [40,41]. Therefore, the ratio of GSH/GSSG is a critical indicator of oxidative stress. In our LPS-induced murine ALI model, the GSH level and the GSH/GSSG ratio appeared to increase after melatonin treatment, suggesting that melatonin possessed a strong antioxidant ability. Melatonin restored the GSH/GSSG ratio to guide the DCs to rebalance the immune network, which might be key to promoting ALI recovery.

In the GSH antioxidant system, there is a group of functionally related enzymes termed glutathione peroxidases (GPxs), which have the ability to use GSH as a substrate to decrease hydroperoxides, including lipid hydroperoxides [42]. In line with the result of the GSH levels, the addition of melatonin in vitro and in vivo was shown to dramatically attenuate GPx enzyme activity. CAT and SOD, as other important antioxidant enzymes, also play a key role in the antioxidant network. CAT can eliminate high levels of H_2_O_2_, and SOD, as the first characterized antioxidant enzyme, can dismutate two O_2_^•−^ anions to H_2_O_2_ and molecular oxygen [43]. These antioxidant enzymes naturally exist in the lungs; however, the intrapulmonary levels of these enzymes can be largely increased when the lung faces high oxidative stress conditions [44]. Our results indicated that melatonin significantly promoted the level of CAT and SOD not only in the LPS-induced murine ALI model in vivo but also in the LPS-induced DCs in vitro, indicating that melatonin possesses the capability to balance oxidative stress by up-regulating antioxidative enzyme activity.

Oxidative stress is broadly linked with the regulation of immune and inflammatory responses, thereby contributing to inflammatory injury [19]. Notably, the elevation of pro-inflammatory cytokines, such as IL-1β and IL-6, is a common characteristic of lung injuries of diverse etiologies [45]. Our previous study indicated that ROS can promote the maturation process of DCs, which remarkably enhanced a large amount of IL-1β and IL-6 secretion for downstream T-cell activation [46]. Meanwhile, IL-1β and IL-6 can disturb the antioxidant enzyme system, resulting in an imbalance of oxidative stress [47]. TGF-β can induce ROS generation [48] and is a critical mediator of pulmonary edema in ALI [49]. In our data, melatonin reduced the production of IL-1β, IL-6, and TGF-β both in the LPS-induced murine ALI model and in LPS-induced DCs, indicating that melatonin, as an antioxidant, can effectively attenuate overloaded cytokine secretion in vivo and in vitro. A previous study suggested that melatonin inhibited the IL-1β-activated NLRP3 inflammasome for inflammatory control [25]. Another team found that melatonin inhibited the p38 MAPK and nuclear factor-κβ (NF-κB) signaling pathway for blocking the expression of pro-inflammatory cytokines [50]. However, the underlying mechanism is still unclear and needs further work.

Nrf2, a transcription factor, is responsible for the regulation of oxidative stress. Various downstream antioxidant enzymes can be regulated upon Nrf2 activation [51]. HO-1, an important Nrf2-related target, can prevent the occurrence and development of ALI via the regulation of oxidative stress [52]. Therefore, the Nrf2/HO-1 axis is a potential activation target against oxidative stress in treatment strategies for ALI. We found that melatonin did not disturb the balance of oxidative stress in the normal state; however, it strongly activated the Nrf2/HO-1 axis in the LPS-induced DCs compared with that in the LPS-only group, which clarified an underlying antioxidant mechanism for melatonin. In addition, our data hinted that LPS up-regulated the expression level of HO-1 in DCs, which is in line with previous studies showing that HO-1 expression can be induced by LPS via activator protein-1 (AP-1) activation in macrophages [53,54]. Of interest, HO-l is commonly induced by various agents that lead to oxidant stress, including hydrogen peroxide, ultraviolet irradiation, and sodium arsenite [55]. However, HO-l activation by LPS is limited, which may be not sufficient to maintain antioxidant homeostasis against LPS-induced oxidative stress. Notably, melatonin strengthens the antioxidant capacity of DCs to correct LPS-induced antioxidant injury.

## 5. Conclusions

In summary, the current study showed a clear antioxidative ability of melatonin in the protection of LPS-induced DCs and an LPS-induced murine ALI model against oxidative stress for inflammatory control, providing the possibility for a DC-targeted oxidative stress regulation strategy for ALI treatment.

## Data Availability

The data are contained within this article.
